# Dataset on aerosol loading, size and statistics over Sanpedro

**DOI:** 10.1016/j.dib.2018.08.117

**Published:** 2018-08-30

**Authors:** M.E. Emetere, J.M. Emetere, T. Osunlola

**Affiliations:** a Department of Physics, Covenant University Canaan land, P.M.B 1023, Ota, Nigeria; b Department of Mathematics, Federal University of Technology, Minna, Nigeria; c Department of Mechanical Engineering and Science, University of Johannesburg, APK, South Africa

**Keywords:** Aerosol loading, Aerosol, Sanpedro, Cote d’Ivoire, Model

## Abstract

Dataset on the aerosol loading and aerosol size radius was provided to guide researcher on the health implication of the air pollution over the research area. Fifteen years primary (aerosol optical depth) dataset was obtained from the Multi-angle Imaging Spectro-Radiometer (MISR). Aerosol loading were generated from the primary dataset. The dataset is important the major cause of respiratory diseases recorded in Sanpedro-Cote d’Ivoire.

**Specifications Table**

**Table:** 

Subject area	Air pollution
More specific subject area	Aerosol loading and Retention,aerosol size and aerosol optical depth statistics
Type of data	Table and figure
How data was acquired	Multi-angle Imaging Spectro-Radiometer (MISR).
Data format	Raw and analyzed
Experimental factors	Data retrieval of Aerosol Optical Depth, data processing and statistics
Experimental features	Measurement at 550 nm
Data source location	Sanpedro-Cote d’Ivoire
Data accessibility	https://l0dup05.larc.nasa.gov/L3Web/download

**Value of the data**

• The data provides the health implication of antropogenic emission in the study area.
• The data provides basis for modelling health hazard on inhalation.
• The data reveals the current state of air pollution.
• The data provides a platform to validate data from emerging models.

## 1 Data

The current air pollution in the research area is alarming and may exceed the World Health Organization estimates if not controlled [1]. The West African regional scale dispersion model has been proven to accurately measure aerosol loading via satellite measurements [2], [3], [4]. The summarized primary data was obtained from Multi-angle Imaging Spectro-Radiometer (MISR) i.e. found in Table 1A, Table 1B, Table 1CA-C for 550 nm wavelength [5]. The aersosl optical depth is found in Table 1A, Table 1B, Table 1CA-C. The empty spaces in Table 1A, Table 1B, Table 1CA-C are the missing data perculiar to West Africa. Ref [6] revealed that the missing data is due to moisture, precipitation rate and cloud cover over West Africa. Aerosol loading over the area was obtained using the West African regional scale dispersion model [6] from the primary dataset (Table 2A, Table 2B, Table 2CA-C). The Angstrom parameter is presented in Table 3A, Table 3B, Table 3C. The radius of particulateusing the back-envelope cateria is shown in Table 4A, Table 4B, Table 4CA-C. The radius of the particulate that determine the level of danger of aerosols by inhalation is presented in Table 5A, Table 5B, Table 5CA-C. The empty spaces in Table 3A, Table 3B, Table 3CA-C to Table 5A, Table 5B, Table 5CA-C is due to missing data that has been explained above. The statistical analysis of the summarized promary dataset is shown in Table 6A, Table 6B, Table 6CA-C.

**Table 1A:** Summarized Aerosol Optical Depth Dataset over Sanpedro for year 2000–2004.

**Month**	**2000**	**2001**	**2002**	**2003**	**2004**
**Jan**	0.743	0.654	0.757	0.678	0.561
**Feb**	0.359	0.817	0.758	0.359	0.838
**Mar**	0.281	0.711	0.575	0.619	0.891
**Apr**		0.257	0.275	0.224	0.476
**May**				0.251	0.360
**Jun**	0.592				
**Jul**	0.328			0.251	
**Aug**	0.185	0.451	0.295	0.414	
**Sep**	0.312	0.221	0.216		
**Oct**	0.394		0.176	0.188	
**Nov**		0.195		0.369	
**Dec**		0.469	0.420	0.550	0.653

**Table 1B:** Summarized Aerosol Optical Depth Dataset over Sanpedro for year 2008–2009.

**Month**	**2005**	**2006**	**2007**	**2008**	**2009**
**Jan**	0.689	0.453	0.867	0.584	0.744
**Feb**	0.619	0.371	0.559	0.916	0.442
**Mar**	0.528	0.605	1.004	0.703	0.412
**Apr**		0.356	0.291	0.306	0.247
**May**		0.151	0.252		0.245
**Jun**					
**Jul**	0.473			0.414	
**Aug**	0.245	0.125		0.332	0.471
**Sep**	0.689	0.357	0.230		0.362
**Oct**		0.312	0.295	0.331	0.212
**Nov**	0.343	0.481	0.275	0.330	0.449
**Dec**	0.567	0.610	0.652	0.564	0.549

**Table 1C:** Summarized Aerosol Optical Depth Dataset over Sanpedro for year 2010–2013.

Month	2010	2011	2012	2013
Jan	0.574	0.795	0.637	0.562
Feb	0.336	0.528	0.666	0.578
Mar	1.505	0.362	0.771	0.359
Apr	0.210		0.294	0.350
May		0.323	0.382	
Jun		0.445	0.402	
Jul		0.350	0.226	
Aug	0.388		0.309	0.479
Sep	0.294	0.317	0.279	
Oct	0.218	0.237	0.308	0.278
Nov	0.189	0.391		
Dec	0.431	0.675	0.442

**Table 2A:** Aerosol loading over Sanpedro between years 2000 and 2004.

**Month**	**2000**	**2001**	**2002**	**2003**	**2004**
**Jan**	0.729	0.774	0.721	0.762	0.817
**Feb**	0.891	0.688	0.721	0.891	0.676
**Mar**	0.911	0.745	0.811	0.791	0.646
**Apr**	0.944	0.916	0.912	0.923	0.851
**May**	0.944	0.944	0.944	0.918	0.890
**Jun**	0.803	0.944	0.944	0.944	0.944
**Jul**	0.899	0.944	0.944	0.918	0.944
**Aug**	0.930	0.861	0.908	0.873	0.944
**Sep**	0.903	0.924	0.925	0.944	0.944
**Oct**	0.880	0.944	0.931	0.929	0.944
**Nov**	0.944	0.928	0.944	0.888	0.944
**Dec**	0.944	0.854	0.871	0.822	0.775

**Table 2B:** Aerosol loading over Sanpedro between years 2005 and 2009.

**Month**	**2005**	**2006**	**2007**	**2008**	**2009**
**Jan**	0.757	0.860	0.660	0.807	0.729
**Feb**	0.791	0.887	0.818	0.631	0.864
**Mar**	0.831	0.797	0.577	0.750	0.874
**Apr**	0.944	0.891	0.909	0.905	0.919
**May**	0.944	0.935	0.918	0.944	0.919
**Jun**	0.944	0.944	0.944	0.944	0.944
**Jul**	0.853	0.944	0.944	0.873	0.944
**Aug**	0.919	0.938	0.944	0.898	0.853
**Sep**	0.757	0.891	0.922	0.944	0.890
**Oct**	0.944	0.904	0.908	0.899	0.925
**Nov**	0.895	0.849	0.912	0.899	0.861
**Dec**	0.814	0.795	0.775	0.816	0.822

**Table 2C:** Aerosol loading over Sanpedro between years 2010 and 2013.

**Month**	**2010**	**2011**	**2012**	**2013**
**Jan**	0.811	0.701	0.782	0.816
**Feb**	0.897	0.831	0.768	0.809
**Mar**	0.268	0.890	0.714	0.891
**Apr**	0.926	0.944	0.908	0.893
**May**	0.944	0.901	0.884	0.944
**Jun**	0.944	0.863	0.877	0.944
**Jul**	0.944	0.893	0.923	0.944
**Aug**	0.882	0.944	0.904	0.850
**Sep**	0.908	0.902	0.912	0.944
**Oct**	0.924	0.921	0.905	0.912
**Nov**	0.929	0.881	0.944	0.944
**Dec**	0.868	0.764	0.864	0.944

**Table 3A:** Angstrom parameter over Sanpedro between years 2000 and 2004.

**Month**	**2000**	**2001**	**2002**	**2003**	**2004**
**Jan**	0.047	0.067	0.044	0.061	0.092
**Feb**	0.162	0.032	0.044	0.162	0.028
**Mar**	0.201	0.054	0.088	0.076	0.018
**Apr**		0.215	0.204	0.237	0.117
**May**				0.219	0.162
**Jun**	0.083				
**Jul**	0.176			0.219	
**Aug**	0.267	0.126	0.193	0.140	
**Sep**	0.184	0.239	0.243		
**Oct**	0.148		0.275	0.265	
**Nov**		0.259		0.158	
**Dec**		0.120	0.137	0.095	0.067

**Table 3B:** Angstrom parameter over Sanpedro between years 2005 and 2009.

**Month**	**2005**	**2006**	**2007**	**2008**	**2009**
**Jan**	0.059	0.125	0.023	0.085	0.047
**Feb**	0.076	0.157	0.092	0.014	0.129
**Mar**	0.101	0.079	-0.001	0.056	0.140
**Apr**		0.163	0.195	0.187	0.222
**May**		0.299	0.218		0.223
**Jun**					
**Jul**	0.119			0.140	
**Aug**	0.223	0.329		0.174	0.119
**Sep**	0.059	0.163	0.233		0.161
**Oct**		0.184	0.193	0.175	0.245
**Nov**	0.169	0.116	0.204	0.175	0.127
**Dec**	0.090	0.078	0.068	0.091	0.095

**Table 3C:** Angstrom parameter over Sanpedro between years 2010 and 2013.

**Month**	**2010**	**2011**	**2012**	**2013**
**Jan**	0.088	0.036	0.071	0.091
**Feb**	0.173	0.101	0.064	0.087
**Mar**	-0.065	0.161	0.041	0.162
**Apr**	0.247		0.194	0.166
**May**		0.179	0.152	
**Jun**		0.128	0.144	
**Jul**		0.166	0.235	
**Aug**	0.150		0.186	0.117
**Sep**	0.194	0.182	0.202	
**Oct**	0.241	0.228	0.187	0.203
**Nov**	0.264	0.149		
**Dec**	0.133	0.062	0.129

**Table 4A:** Radius of particulate-back of envelope calculation between years 2000 and 2004.

**Month**	**2000**	**2001**	**2002**	**2003**	**2004**
**Jan**	0.553	0.533	0.555	0.539	0.511
**Feb**	0.451	0.567	0.555	0.451	0.571
**Mar**	0.422	0.546	0.514	0.525	0.581
**Apr**		0.411	0.419	0.396	0.488
**May**				0.409	0.452
**Jun**	0.519				
**Jul**	0.440			0.409	
**Aug**	0.375	0.481	0.427	0.470	
**Sep**	0.434	0.394	0.392		
**Oct**	0.463		0.370	0.377	
**Nov**		0.381		0.455	
**Dec**		0.486	0.471	0.508	0.533

**Table 4B:** Radius of particulate-back of envelope calculation between years 2005 and 2009.

**Month**	**2005**	**2006**	**2007**	**2008**	**2009**
**Jan**	0.541	0.481	0.577	0.517	0.553
**Feb**	0.525	0.456	0.510	0.585	0.478
**Mar**	0.502	0.522	0.601	0.544	0.469
**Apr**		0.450	0.426	0.432	0.406
**May**		0.355	0.409		0.406
**Jun**					
**Jul**	0.487			0.470	
**Aug**	0.406	0.337		0.442	0.487
**Sep**	0.541	0.450	0.399		0.452
**Oct**		0.434	0.427	0.441	0.390
**Nov**	0.446	0.490	0.419	0.441	0.480
**Dec**	0.512	0.523	0.533	0.512	0.508

**Table 4C:** Radius of particulate-back of envelope calculation between years 2010 and 2013.

**Month**	**2010**	**2011**	**2012**	**2013**
**Jan**	0.514	0.563	0.529	0.511
**Feb**	0.443	0.502	0.536	0.515
**Mar**	0.672	0.452	0.558	0.451
**Apr**	0.389		0.427	0.448
**May**		0.438	0.459	
**Jun**		0.479	0.466	
**Jul**		0.448	0.397	
**Aug**	0.461		0.433	0.489
**Sep**	0.427	0.436	0.421	
**Oct**	0.393	0.402	0.432	0.420
**Nov**	0.378	0.462		
**Dec**	0.475	0.538	0.478

**Table 5A:** Radius of particulate-atmospheric aerosols between years 2000 and 2004.

**Month**	**2000**	**2001**	**2002**	**2003**	**2004**
**Jan**	7.11E-07	6.68E-07	7.17E-07	6.79E-07	6.22E-07
**Feb**	5.16E-07	7.46E-07	7.17E-07	5.16E-07	7.55E-07
**Mar**	4.71E-07	6.95E-07	6.29E-07	6.51E-07	7.80E-07
**Apr**		4.56E-07	4.67E-07	4.34E-07	5.79E-07
**May**				4.52E-07	5.17E-07
**Jun**	6.37E-07				
**Jul**	4.99E-07			4.52E-07	
**Aug**	4.06E-07	5.66E-07	4.79E-07	5.46E-07	
**Sep**	4.89E-07	4.32E-07	4.28E-07		
**Oct**	5.35E-07		4.00E-07	4.09E-07	
**Nov**		4.14E-07		5.22E-07	
**Dec**		5.75E-07	5.50E-07	6.17E-07	6.67E-07

**Table 5B:** Radius of particulate-atmospheric aerosols between years 2005 and 2009.

**Month**	**2005**	**2006**	**2007**	**2008**	**2009**
**Jan**	7.71E-07	5.67E-07	7.69E-07	6.33E-07	7.11E-07
**Feb**	7.55E-07	5.23E-07	6.21E-07	7.92E-07	5.61E-07
**Mar**	7.32E-07	6.44E-07	8.33E-07	6.91E-07	5.45E-07
**Apr**		5.15E-07	4.77E-07	4.86E-07	4.49E-07
**May**		3.80E-07	4.52E-07		4.48E-07
**Jun**					
**Jul**	7.16E-07			5.46E-07	
**Aug**	6.33E-07	3.58E-07		5.01E-07	5.76E-07
**Sep**	7.71E-07	5.15E-07	4.38E-07		5.18E-07
**Oct**		4.89E-07	4.79E-07	5.00E-07	4.26E-07
**Nov**	6.74E-07	5.82E-07	4.67E-07	5.00E-07	5.65E-07
**Dec**	7.42E-07	6.46E-07	6.67E-07	6.24E-07	6.16E-07

**Table 5C:** Radius of particulate-atmospheric aerosols between years 2010 and 2013.

**Month**	**2010**	**2011**	**2012**	**2013**
**Jan**	6.29E-07	7.35E-07	6.60E-07	6.23E-07
**Feb**	5.03E-07	6.05E-07	6.74E-07	6.31E-07
**Mar**	1.07E-06	5.18E-07	7.24E-07	5.16E-07
**Apr**	4.25E-07		4.79E-07	5.11E-07
**May**		4.96E-07	5.29E-07	
**Jun**		5.63E-07	5.40E-07	
**Jul**		5.11E-07	4.35E-07	
**Aug**	5.32E-07		4.88E-07	5.80E-07
**Sep**	4.79E-07	4.92E-07	4.69E-07	
**Oct**	4.30E-07	4.43E-07	4.87E-07	4.69E-07
**Nov**	4.10E-07	5.34E-07		
**Dec**	5.55E-07	6.78E-07	5.61E-07

**Table 6A:** AOD statistics over Sanpedro between years 2000 and 2004.

**Statistics**	**2000**	**2001**	**2002**	**2003**	**2004**
Number of values	8.000	8.000	8.000	10.000	6.000
Number of missing values	4.000	4.000	4.000	2.000	6.000
Mean	0.399	0.472	0.434	0.390	0.630
First quartile	0.297	0.239	0.245	0.251	0.476
Third quartile	0.493	0.682	0.666	0.550	0.838
Standard error	0.064	0.084	0.083	0.055	0.084
95% confidence interval	0.152	0.199	0.197	0.124	0.217
99% confidence interval	0.224	0.294	0.291	0.178	0.340
Variance	0.033	0.057	0.056	0.030	0.043
Average deviation	0.134	0.192	0.197	0.140	0.164
Standard deviation	0.181	0.238	0.236	0.173	0.207
Coefficient of variation	0.454	0.504	0.543	0.444	0.328
Skew	1.129	0.199	0.529	0.575	0.108
Kurtosis	0.760	−1.622	−1.556	−1.081	−1.475
Kolmogorov-Smirnov stat	0.262	0.191	0.223	0.189	0.177
Critical K-S stat, alpha = 0.10	0.410	0.410	0.410	0.369	0.468
Critical K-S stat, alpha = 0.05	0.454	0.454	0.454	0.409	0.519
Critical K-S stat, alpha = 0.01	0.542	0.542	0.542	0.489	0.617

**Table 6B:** AOD statistics over Sanpedro between years 2005 and 2009.

**Statistics**	**2005**	**2006**	**2007**	**2008**	**2009**
Number of values	8.000	10.000	9.000	9.000	10.000
Number of missing values	4.000	2.000	3.000	3.000	2.000
Minimum	0.519	0.382	0.492	0.498	0.413
First quartile	0.408	0.312	0.269	0.330	0.247
Third quartile	0.654	0.481	0.706	0.613	0.471
Standard error	0.056	0.052	0.098	0.070	0.051
95% confidence interval	0.134	0.117	0.225	0.162	0.115
99% confidence interval	0.198	0.169	0.327	0.236	0.165
Variance	0.025	0.027	0.086	0.044	0.026
Average deviation	0.124	0.124	0.248	0.172	0.118
Standard deviation	0.160	0.164	0.293	0.211	0.161
Coefficient of variation	0.308	0.429	0.596	0.424	0.389
Skew	−0.709	−0.171	0.860	1.047	0.711
Kurtosis	−0.479	−0.563	−0.822	0.317	0.745
Kolmogorov-Smirnov stat	0.146	0.138	0.305	0.228	0.159
Critical K-S stat, alpha =0.10	0.410	0.369	0.387	0.387	0.369
Critical K-S stat, alpha =0.05	0.454	0.409	0.430	0.430	0.409
Critical K-S stat, alpha =0.01	0.542	0.489	0.513	0.513	0.489

**Table 6C:** AOD statistics over Sanpedro between years 2010 and 2013.

**Statistics**	**2010**	**2011**	**2012**	**2013**
Number of values	9.000	10.000	11.000	6.000
Number of missing values	3.000	2.000	1.000	6.000
Mean	0.460	0.442	0.429	0.434
First quartile	0.216	0.323	0.297	0.350
Third quartile	0.467	0.528	0.588	0.562
Standard error	0.137	0.055	0.055	0.050
95% confidence interval	0.315	0.125	0.122	0.130
99% confidence interval	0.459	0.180	0.174	0.203
Variance	0.168	0.031	0.033	0.015
Average deviation	0.257	0.135	0.146	0.105
Standard deviation	0.410	0.175	0.182	0.124
Coefficient of variation	0.891	0.396	0.424	0.284
Skew	2.516	1.122	0.909	0.047
Kurtosis	6.775	0.502	-0.527	-2.091
Kolmogorov-Smirnov stat	0.306	0.216	0.200	0.229
Critical K-S stat, alpha = 0.10	0.387	0.369	0.352	0.468
Critical K-S stat, alpha = 0.05	0.430	0.409	0.391	0.519
Critical K-S stat, alpha = 0.01	0.513	0.489	0.468	0.617

## 2 Experimental design, materials and methods

**Fig. 1:**
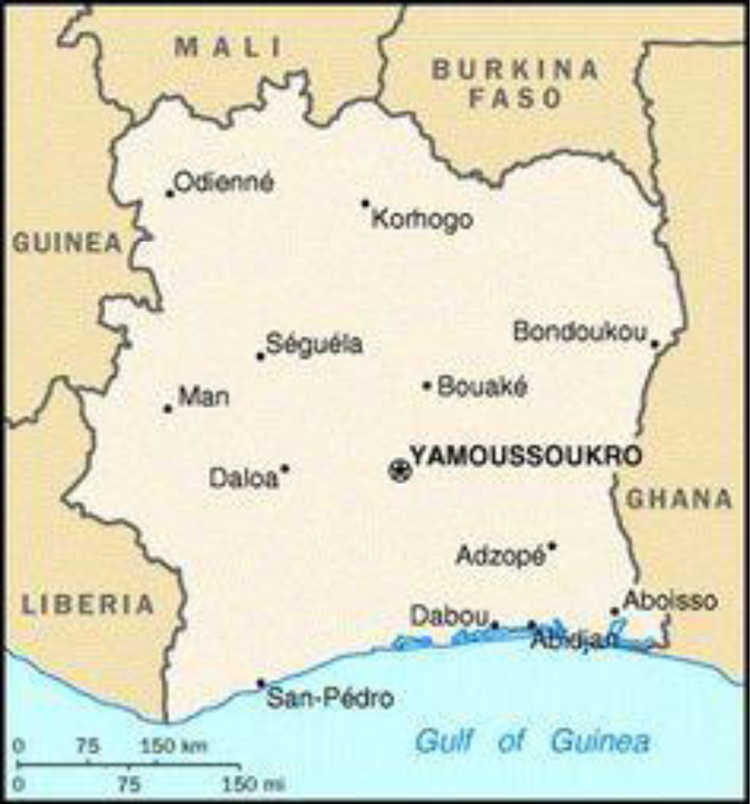
Geographical map of Sanpedro.

Sanpedro is located in Cote d’Ivoire on longitude and latitude of 6.6424 °W and 4.7579 °N (Fig. 1).

The West African regional scale dispersion model (WASDM) for calculating aerosol loading over a region [6]:

(1) $$\psi \left(\lambda \right)={a_{1}}^{2}\cos\left(\frac{n_{1}\pi \tau (\lambda )}{2}x\right)\cos\left(\frac{n_{1}\pi \tau (\lambda )}{2}y\right)+\ldots \ldots {a_{n}}^{2}\cos\left(\frac{n_{n}\pi \tau (\lambda )}{2}x\right)\cos\left(\frac{n_{n}\pi \tau (\lambda )}{2}y\right)$$

a is atmospheric constant gotten from the fifteen years aerosol optical depth (AOD) dataset from MISR, n is the tunning constant, τ(λ) is the AOD of the area and ψ(λ) is the aerosol loading. The data processing was done using the excel. The validation of the summarized dataset was done using mathematical models and statistical softwares. The analysis of Eq. (1) was done using the C++ codes.
